# Resident physician training in bedside pleural procedures: A one-year experience at a teaching hospital

**DOI:** 10.1016/j.clinsp.2024.100399

**Published:** 2024-06-03

**Authors:** Diego Arley Gomes da Silva, Paula Duarte D'Ambrosio, Fabio Eiti Nishibe Minamoto, Bernardo Mulinari de Lacerda Pessoa, Eserval Rocha Junior, Leticia Leone Lauricella, Ricardo Mingarini Terra, Paulo Manuel Pêgo-Fernandes, Alessandro Wasum Mariani

**Affiliations:** a Divisao Cirurgia Toracica, Instituto do Coracao, Hospital das Clinicas HCFMUSP, Faculdade de Medicina, Universidade de Sao Paulo, Sao Paulo, SP, Brazil; b Instituto do Câncer, Hospital das Clínicas, Faculdade de Medicina, Universidade de São Paulo (HCFMUSP), São Paulo, SP, Brazil

**Keywords:** Pleural diseases, Thoracentesis, Thoracic drainage, Minor surgical procedures, Medical education

## Abstract

**Background and objective:**

This study aims to quantify bedside pleural procedures performed at a quaternary teaching hospital describing technical and epidemiological aspects.

**Materials and methods:**

The authors retrospectively reviewed consecutive patients who underwent invasive thoracic bedside procedures between March 2022 and February 2023.

**Results:**

463 chest tube insertions and 200 thoracenteses were performed during the study period. Most procedures were conducted by 1st-year Thoracic Surgery residents, with Ultrasound Guidance (USG). There was a notable preference for small-bore pigtail catheters, with a low rate of immediate complications.

**Conclusion:**

Bedside thoracic procedures are commonly performed in current medical practice and are significant in surgical resident training. The utilization of pigtail catheters and point-of-care ultrasonography by surgical residents in pleural procedures is increasingly prevalent and demonstrates high safety.

## Highlights

• Thoracentesis and chest tube insertions are frequent procedures in the medical routine.
• Resident physician training includes practice in pleural procedures.
• Point-of-care ultrasound is indicated when performing these procedures.
• Using small-bore pigtail pleural catheters is safe and increasingly common.

## Introduction

Pleural pathologies frequently require invasive procedures for diagnosis and treatment.^1^ Non-specialized practitioners, surgical specialties, or thoracic surgeons can perform these procedures. However, they carry inherent risks that can lead to significant morbidity and mortality.^2^ Therefore, physicians and surgeons in training must receive proper education and training to perform such pleural procedures ensuring efficacy and safety.^3^

The most performed bedside pleural procedures are thoracentesis, or pleural tap, and chest tube placement. Both procedures are essential in various clinical scenarios, including pleural effusions, pneumothorax, and hemothorax, making them a vital component of surgical trainees' education and training.^4^^,^^5^

The techniques and equipment used for these procedures can vary, and there is an ongoing discussion in the literature regarding the optimal approaches and tube models. Factors such as real-time visualization and tube caliber selection contribute to the complexity of these procedures.^6^, ^7^, ^8^, ^9^

To achieve better outcomes in bedside pleural procedures, it is recommended that institutions and healthcare services establish standardized training protocols. This includes defining minimum competency levels before trainees are allowed to perform these procedures independently.^4^

In this study, the authors aim to quantify the frequency of bedside pleural procedures and comprehensively analyze their epidemiological and technical aspects, including immediate complications rate, in Hospital das Clinicas, Faculdade de Medicina, Universidade de São Paulo (HCFMUSP), the largest quaternary teaching hospital in Latin America.

## Materials and methods

This study employed a descriptive, retrospective observational design using convenience sampling of all consecutive procedures. The authors reviewed our prospective electronic database for thoracic procedures and completed the information when needed by reviewing the electronic medical records of patients submitted to thoracentesis or chest tube placement procedures performed by the thoracic surgery team at the Hospital das Clínicas da Faculdade de Medicina da Universidade de São Paulo (HCFMUSP). The study period spanned from March 2022 to February 2023 and included patients linked to the institution's medical residency programs. Patients with incomplete information regarding the procedures were excluded from the analysis.

Data was collected by extracting information from our prospective electronic database for thoracic procedures standardized on the REDCap platform®, where all procedures under the responsibility of the Hospital's Thoracic Surgery team are prospectively recorded, with previous approval from the institution's research ethics committee; in case of incomplete information, a second investigator reviewed the procedure reports and clinical notes documented in conventional institutional electronic medical records. Patients who had incomplete or non-existent data after the second reviewer's assessment were excluded from registration on the REDCap platform. Subsequently, the collected data were tabulated using the IBM SPSS Statistics 21® descriptive statistics tool, enabling the calculation of frequencies, percentages, means, and Standard Deviations (SD). The data were then organized into tables for analysis and presentation.

The project was submitted for evaluation by the Research Ethics Committee of the institution, approved under certificate (CAAE) number 4.170.936.

## Results

Between March 2022 and February 2023, 663 bedside procedures were performed by the Thoracic Surgery team of HCFMUSP. Among these procedures, 463 (69.8 %) chest tube placements, while 200 (30.2 %) thoracenteses. The mean age of the sample was 57.2 years (± 17.47 SD), with a predominance male population accounting for 54.8 % of the cases. Please refer to Table 1 for a detailed description of the sample characteristics. Immediate complications were reported in 21 procedures (3.2 %), as outlined in Table 2.

**Table 1:** Comparison between pleural drainages and thoracentesis performed between March 2022 and February 2023 at HCFMUSP (SD, Standard Deviation).

	Chest tube placement (*n* = 463)	Thoracentesis (*n* = 200)
**Age**	55.5 (± 17.76 SD)	61.5 (± 16.02 SD)
**Sex**		
Male	267 (57.7 %)	96 (48.0 %)
Female	196 (42.3 %)	104 (52.0 %)
**ECOG-PS**		
0	25 (5.4 %)	9 (4.5 %)
1	68 (14.7 %)	45 (22.5 %)
2	115 (24.8 %)	77 (38.5 %)
3	99 (21.4 %)	43 (21.5 %)
4	156 (33.7 %)	26 (13.0 %)
**Location**		
ICU	201 (43.4 %)	28 (14.0 %)
Wards	133 (28.7 %)	90 (45.0 %)
Emergency Room	32 (6.9 %)	7 (3.5 %)
Urgent Care	75 (16.2 %)	52 (26.0 %)
Outpatient	10 (2.2 %)	19 (9.5 %)
Clinics	8 (1.7 %)	4 (2.0 %)
Post Anesthesia Care Unit	4 (0.9 %)	0 (0.0 %)
**Performed by**		
1st year resident (general or cardiac surgery, emergency medicine)	141 (30.5 %)	94 (47.0 %)
1st year thoracic surgery resident	310 (67.0 %)	106 (53.0 %)
2nd year thoracic surgery resident	5 (1.1 %)	0 (0.0 %)
Attending surgeon	6 (1.3 %)	0 (0.0 %)
Procedure site		
Right side	267 (57.7 %)	112 (56.0 %)
Left side	189 (40.8 %)	86 (42.5 %)
Bilateral	7 (1.5 %)	3 (1.5 %)

SD, Standard Deviation; ECOG-PS, Eastern Cooperative Oncology Group Performance Status Scale; ICU, Intensive Care Unit.

**Table 2:** Immediate complications in pleural procedures between March 2022 and February 2023 at HCFMUSP.

Immediate complication	Chest tube placement (*n* = 463)	Thoracentesis (*n* = 200)
None	448 (96.8 %)	194 (97.0 %)
Yes	15 (3.2 %)	7 (3.0 %)
Bleeding	0	1 (0.5 %)
Syncope, lipothymia, dyspnoea, or re-expansion pulmonary edema	6 (1.1 %)	2 (1.0 %)
Cardiopulmonary arrest	1 (0.2 %)	0
Malpositioning	0	0
Severe hypoxemia	1 (0.2 %)	0
Pneumothorax	1 (0.2 %)	1 (0.5 %)
Ineffective drainage	6 (1.1 %)	3 (1.5 %)

Among the 463 bedside chest tube placements for pleural procedures, the mean average age of patients was 56.3 years (± 17.66 SD), with 57.7 % male. Most patients 255 (55.1 %) had a poor performance status (Eastern Cooperative Oncology Group Performance Status^10^ ‒ ECOG-PS 3 or 4). These chest tube insertions were mainly performed in an Intensive Care Unit (ICU) and ward settings, predominantly by first-year Thoracic Surgery trainees, under the supervision of the senior fellow or attending surgeon specialized in this field (Table 1).

The main indication for chest tube insertions in our sample was pleural effusion of undetermined etiology, followed by pneumothorax (Table 3). Using 14 French (Fr) pigtail pleural catheters was more prevalent (90.5 %) than procedures involving larger bore tubular drains (9.5 %). USG was employed in 66.1 % of pleural drainages for site demarcation and/or guidance during the procedure.

**Table 3:** Clinical indications of pleural procedures between March 2022 and February 2023 at HCFMUSP.

Main indication	Chest tube placement (*n* = 463)	Thoracentesis (*n* = 200)
Pneumothorax	147 (31.7 %)	0
Pleural effusion without established etiology before the procedure	187 (40.4 %)	150 (75.0 %)
Heart failure/transudate	11 (2.4 %)	22 (11.0 %)
Recurrent malignant pleural effusion	59 (12.7 %)	26 (13.0 %)
Chylothorax	10 (2.2 %)	0
Pleural empyema	34 (7.3 %)	2 (1.0 %)
Hemothorax	14 (3.0 %)	0

Regarding thoracocentesis, the average age was 61.5 years (± 16.02 SD), with a slightly higher proportion of female patients (52.0 %) and better performance status (ECOG-PS 1 or 2) compared to those undergoing chest tube placement (Table 1). The majority of the thoracentesis was performed in the ward setting (45.0 %) and by first-year specialty residents (53.0 %) under the supervision of a higher-level resident or assistant.

Ultrasound was employed in 89.5 % of thoracentesis, and the average volume of fluid aspirated in our sample was 747.4 ± 457.58 mL (mean ± SD). Only two patients (1.1 %) underwent thoracentesis under invasive mechanical ventilation during the procedure.

## Discussion

Thoracic procedures are frequent in medical practice, whose form of teaching and training is classically linked to the apprentice model of “see, do, teach”,^11^ based on direct experimentation in the clinical environment,^12^ with a scarcity of data on the performance of resident physicians in their execution.^13^

Recently, the European Society of Thoracic Surgeons (ESTS) formed a task force to develop a consensus on the competencies expected in the training of Thoracic Surgeons in Europe. The document highlights the need for high-quality training programs that cover all the aspects mentioned. The skills assessed include bedside procedures, particularly pleural procedures, whose assessments can be carried out in intensive care units and surgical centers, preferably with dedicated tools with evidence of validity.^14^

A survey with 34 key opinion leaders in thoracic surgery from worldwide listed chest tube insertion as one of the 17 essential procedures that a newly qualified thoracic surgeon should be able to perform based on parameters such as frequency, risk of the procedure and feasibility of simulation-based education.^15^

In our practice, these procedures were predominantly performed by 1st-year thoracic surgery residents, followed by residents of other specialties whose training program includes a Thoracic Surgery rotation. The authors encouraged and taught all residents, unconstrained of the specialty, how to properly use ultrasound to perform these procedures,^16^ to minimize the risk of complications and improve the quality of care.^1^

Although performed at the bedside, all procedures were performed under maximum sterile precautions, using a sterile apron, gloves, cap, and total body cover, according to our institutional recommendation for invasive procedures.^4^

Ultrasound-guided thoracentesis and small-bore pigtail pleural catheter placement are associated with a low rate of complications.^17^ A systematic review of articles published between 2010 and 2021 identified 156,810 thoracentesis and 4,816 pleural drainages.^18^ Among the thoracentesis evaluated in the review, the most frequent complication was pneumothorax (3.3 %), followed by bleeding (1.7 %). At the same time, pulmonary re-expansion edema, which is difficult to define, was reported in 0.1 % of cases.^18^ In chest tube placements, the frequency of bleeding was 1.0 %, and device obstruction was present in 6.3 % of evaluated patients.^18^ In our sample, there was a record of pneumothorax and another of bleeding in the procedures described, both occurred in thoracentesis, but data on complications in these procedures may present inconsistencies due to different methodologies and the need for large samples for identification.^18^^,^^19^

Using Ultrasonography (USG) in performing thoracentesis was frequent in our sample. The use of USG in thoracentesis is recommended in all procedures, as it increases the yield and reduces the risk of complications, especially pneumothorax^19^ and inadvertent visceral puncture, without reducing the incidence of hemothorax due to injury to intercostal vessels, as they are not visible to the method.^5^

USG can be used to mark the puncture site immediately before the procedure, or for real-time monitoring of device insertion, with no controlled studies evaluating the latter strategy.^20^^,^^21^

It is believed that using USG in real-time can minimize the risk of visceral and parenchymal lesions due to the possibility of continuous monitoring with visualization of the needle tip but with the disadvantage of less availability and greater need for training.^21^

In many cases, performing ultrasonography immediately before pleural procedures can change the preferred puncture site, even its indication, or abort its performance.^22^

There was less use of ultrasonography at the bedside in pleural drainages, compared to thoracentesis in our sample, probably due to the high frequency of pneumothorax motivating drainage. Thoracic ultrasound has limited usefulness in guiding pneumothorace drainage due to the difficulty in obtaining images due to the low transmission of sound waves through the air.^5^

The most used pleural drain in the period studied was the 14 Fr diameter pigtail catheter inserted using the trocar technique. These drains can be inserted percutaneously at the bedside.^23^ Historically, the most used chest drains were high bore tubes inserted by dissection technique. Recently, smaller caliber catheters have been popularized, most inserted by “Trocar” or the Seldinger technique.^5^ Some reasons for the tremendous historical use of larger bore tubular drains are the more significant experience in their handling and the higher cost of the pigtail catheter.^24^

Smaller caliber drains have a lower risk of serious complications, less pain, a smaller scar, and less traumatic insertion, being recommended for spontaneous and iatrogenic pneumothorax, and for a diversity of pleural effusions.^5^^,^^8^^,^^9^^,^^25^ The latest ERS/ESTS statement on the management of pleural infection in adults recommends the ultrasound-guided small-bore catheter (12‒14 Fr) as a first-line intervention in pleural infection, with insertion using radiological guidance (ultrasound or computed tomography) and with regular saline flushes.^26^

Drainage of thicker liquids such as hemothorax and later stages of pleural empyema can also be performed with smaller caliber devices.^5^^,^^7^ Its disadvantage can be a greater probability of obstruction by clots or torsion of the device.^8^ However, recent studies comparing tube drains with the 14 Fr pigtail in complicated effusions showed no difference between drainage time, antibiotic therapy, and hospital stay.^9^

The pigtail catheter is also an alternative in the palliative treatment of malignant pleural effusion without compromising the performance of pleurodesis.^24^ Compared to tubular drains, the cost-effectiveness of bedside drainage with a pigtail catheter makes it the most desirable percutaneous device in most clinical settings.^6^

The limitations of our study are the retrospective nature and the absence of comparisons. Regarding the retrospective character, the recall bias should have been minimized since most of the data was collected prospectively in our standardized database. In order to exemplify real-world data, the authors did not carry out any interventions such as modification on the resident training or new techniques in this cohort.

The data refer to the 12-month interval that began two years after the first case of COVID-19 in Brazil, in a period in which the institution's routine practically returned to pre-pandemic parameters. Furthermore, during the first years of the COVID-19 pandemic, the greatest impact occurred on elective non-oncological surgeries, as demonstrated in works from other surgical areas,^27^ but it is noteworthy that bedside pleural procedures are generally performed in acute and subacute settings.^18^ Therefore, the authors believe that there was no significant impact on the routine and number of pleural procedures during the period evaluated.

This paper can help to understand the status of the resident training on bedside invasive pleural procedures regarding current efficiency and safety. The authors believe that different studies are needed to better understand and develop improved techniques for medical training in bedside pleural procedures.

## Conclusion

Bedside thoracic procedures have become an integral part of modern medical practice due to the high prevalence of pleural pathologies. Our data suggest that thoracentesis and chest tube placements can safely be performed by first-year Thoracic Surgery trainees and residents of other specialties under senior supervision with well-established criteria and requirements for their execution, particularly in hospitals of greater complexity and with associated teaching. Using pigtail drainage and point-of-care ultrasound seams to guarantee good accuracy and safety. More data regarding different strategies for teaching Bedside thoracic procedures are needed. New prospective studies, preferably multicentric, are desirable, in order to evaluate the learning curve of bedside procedures performed by training doctors over longer periods, while at the same time reinforcing the need to comply with ethical standards when carrying out such research.

## Human/animal ethics approval declaration

The project was submitted for evaluation by the Research Ethics Committee of the institution, approved under certificate (CAAE) number 4.170.936.

## Funding

This research did not receive any specific grant from funding agencies in the public, commercial, or not-for-profit sectors.

## Declaration of competing interest

The authors declare no conflicts of interest.

## Data Availability

The data that support the findings of this study are available on request from the corresponding author. The data are not publicly available due to privacy or ethical restrictions.
